# Acoustic analysis in stuttering: a machine-learning study

**DOI:** 10.3389/fneur.2023.1169707

**Published:** 2023-06-30

**Authors:** Francesco Asci, Luca Marsili, Antonio Suppa, Giovanni Saggio, Elena Michetti, Pietro Di Leo, Martina Patera, Lucia Longo, Giovanni Ruoppolo, Francesca Del Gado, Donatella Tomaiuoli, Giovanni Costantini

**Affiliations:** ^1^Department of Human Neurosciences, Sapienza University of Rome, Rome, Italy; ^2^IRCCS Neuromed Institute, Pozzilli, Italy; ^3^Department of Neurology, James J. and Joan A. Gardner Center for Parkinson’s Disease and Movement Disorders, University of Cincinnati, Cincinnati, OH, United States; ^4^Department of Electronic Engineering, University of Rome Tor Vergata, Rome, Italy; ^5^CRC – Centro Ricerca e Cura, Rome, Italy; ^6^Department of Sense Organs, Otorhinolaryngology Section, Sapienza University of Rome, Rome, Italy; ^7^IRCCS San Raffaele Pisana, Rome, Italy

**Keywords:** stuttering, machine-learning, telemedicine, home environment, acoustic analysis

## Abstract

**Background:**

Stuttering is a childhood-onset neurodevelopmental disorder affecting speech fluency. The diagnosis and clinical management of stuttering is currently based on perceptual examination and clinical scales. Standardized techniques for acoustic analysis have prompted promising results for the objective assessment of dysfluency in people with stuttering (PWS).

**Objective:**

We assessed objectively and automatically voice in stuttering, through artificial intelligence (i.e., the support vector machine – SVM classifier). We also investigated the age-related changes affecting voice in stutterers, and verified the relevance of specific speech tasks for the objective and automatic assessment of stuttering.

**Methods:**

Fifty-three PWS (20 children, 33 younger adults) and 71 age−/gender-matched controls (31 children, 40 younger adults) were recruited. Clinical data were assessed through clinical scales. The voluntary and sustained emission of a vowel and two sentences were recorded through smartphones. Audio samples were analyzed using a dedicated machine-learning algorithm, the SVM to compare PWS and controls, both children and younger adults. The receiver operating characteristic (ROC) curves were calculated for a description of the accuracy, for all comparisons. The likelihood ratio (LR), was calculated for each PWS during all speech tasks, for clinical-instrumental correlations, by using an artificial neural network (ANN).

**Results:**

Acoustic analysis based on machine-learning algorithm objectively and automatically discriminated between the overall cohort of PWS and controls with high accuracy (88%). Also, physiologic ageing crucially influenced stuttering as demonstrated by the high accuracy (92%) of machine-learning analysis when classifying children and younger adults PWS. The diagnostic accuracies achieved by machine-learning analysis were comparable for each speech task. The significant clinical-instrumental correlations between LRs and clinical scales supported the biological plausibility of our findings.

**Conclusion:**

Acoustic analysis based on artificial intelligence (SVM) represents a reliable tool for the objective and automatic recognition of stuttering and its relationship with physiologic ageing. The accuracy of the automatic classification is high and independent of the speech task. Machine-learning analysis would help clinicians in the objective diagnosis and clinical management of stuttering. The digital collection of audio samples here achieved through smartphones would promote the future application of the technique in a telemedicine context (home environment).

## 1. Introduction

Stuttering is a persistent childhood-onset neurodevelopmental disorder affecting speech fluency (1), typically manifested in 30–48 months old children, and affecting 5%–8% of preschool children (M:F ratio = 1.5:1) (2). Clinically, people with stuttering (PWS) manifest “dysfluency,” namely the deterioration of speech fluency, with a variable association of involuntary, audible, or silent repetitions or prolongations of sounds, syllables, words, sentences, dysrhythmic phonation, such as blocks and prolongations, and broken words (1, 3). PWS may not be able to readily control the impaired vocalization and may also manifest involuntary movements and emotions including fear, embarrassment, or irritation (4–6). The complexity of stuttering behavior tends to change with age, and seems independent from sex, anxiety, or from its intrinsic clinical severity (7). Indeed, stuttering improves with physiologic ageing, thus reducing the prevalence of stuttering at 1% of younger adults starting from the age of 15 (M:F ratio = 4:1) (2, 8, 9). It has been suggested that functional changes in the phonatory apparatus occurring during puberty may play a role in the age-related reduction of stuttering (7). Stuttering is a complex and multifactorial neurodevelopmental disorder whose pathophysiologic mechanisms are supposed to rely on the impairment of several neural networks underlying speech, language, and emotional functions (8, 10).

Currently, the diagnosis of stuttering is based on neuropsychologic (i.e., perceptual) clinical examination with the aid of dedicated clinical scales for the assessment of additional developmental disorders (4, 11, 12). However, clinical scales are qualitative tools which rely on the examiner’s skills and experiences, thus potentially biasing the assessment’s accuracy. To overcome these limitations, as an objective tool for assessing stuttering several authors have adopted acoustic analysis and reported several changes in specific features (13–26). Previous acoustic analysis, however, did not allow to detect the severity of stuttering and monitor its progression. Hence, novel approaches are required for a thorough objective evaluation and assessment of dysfluencies in PWS, at different ages of life.

More recently, artificial intelligence has demonstrated its potential utility in the objective assessment of the human voice under several physiologic and neurologic conditions (27) including laryngeal dystonia, essential tremor, and Parkinson’s disease (28–34). To date, only preliminary data have been reported in PWS, by using machine-learning algorithms, thus showing promising but still preliminary and heterogeneous results. These studies applied automatic stuttering identification systems (ASIS) to a heterogenous dataset of audio samples without a thorough clinical characterization (35–37). Also, most of the previous studies have not considered the effect of ageing on acoustic features in stuttering. Despite a few clinical investigations of stuttering relative to ageing (38), a detailed data-driven analysis in different age-related groups in PWS has never been conducted, so far. Furthermore, none of the previous studies in the field have thoroughly assessed the detrimental effect of linguistic issues, namely worsening of speech in response to specific linguistic tasks. Finally, previous studies on machine-learning in stuttering have not used devices for audio recordings in an ecological scenario.

In the present study, we applied an acoustic analysis based on support vector machine (SVM) classifier to detect abnormal acoustic features in stuttering with the aim of helping clinicians in the automatic and objective classification of stuttering. For this purposes, a large cohort of PWS and controls underwent a thorough clinical investigation, including dedicated clinical scales. The second aim of the study was to assess the effect of ageing on stuttering. Therefore, our cohort included two age-related groups: children (7–12 years old), and young adults (15–30 years old). A further aim of the study was to verify the detrimental effect of linguistic issues on stuttering. Accordingly specific speech tasks have been recorded for each participant. Finally, we aimed to assess the usefulness of machine learning analysis for telemedicine purposes, by recording audio samples with commonly available devices, in an ecological scenario. The sensitivity, specificity, positive/negative predictive values, and accuracy of all diagnostic tests were assessed in detail. Furthermore, we calculated the area under the receiver operating characteristic (ROC) curves to verify the optimal diagnostic threshold as reflected by the associated criterion (Ass. Crit.) and Youden Index (YI). Finally, clinical scale outcomes were correlated with the likelihood ratios (LRs), continuous numerical values providing a measure of stuttering severity for each patient as calculated through feed-forward artificial neural network (ANN) analysis.

## 2. Materials and methods

### 2.1. Participants

We recruited a cohort of 53 people with stuttering (24 females, 29 males; mean age ± SD 16.7 ± 7.6 years, range 7–30) and a group of 71 age- and sex-matched controls (29 females, 44 males; mean age ± SD 16.2 ± 6.5 years, range 7–30). Participants or their legal guardians gave consent to the study, which was approved by the local Institutional Review Board (0026508/2019), following the Declaration of Helsinki. Depending on the age, PWS were included into two independent sex-matched subgroups: 20 children in prepubertal age (cPWS) (9 females, 11 males; mean age ± SD 9.1 ± 1.6 years, range 7–12), and 33 young adults in post-pubertal age (yPWS) (15 females, 18 males; mean age ± SD 21.3 ± 5.8 years, range 15–30). Similarly, 31 controls were included in the subgroup of children (cC) (12 females, 19 males; mean age ± SD 9.6 ± 1.5 years, range 7–12), and in the subgroup of 40 young adults (yC) (17 females, 23 males; mean age ± SD 21.1 ± 3.9 years, range 15–30). Participants were recruited at “*Centro Ricerca e Cura Balbuzie – Disturbi del Linguaggio e dell’Apprendimento*” in Rome, Italy. All participants were native Italian speakers, and non-smokers, and did not manifest cognitive or mood impairment, unilateral/bilateral hearing loss, respiratory disorders, and other disorders potentially affecting the vocal cords. None of the participants was taking drugs acting on the central nervous system at the time of the study. Demographic and anthropometric parameters were collected during the enrollment visit. Also, symptoms related to stuttering were scored using the Italian validated version of the Voice Handicap Index (VHI) (11, 39), the Stuttering Severity Instrument (SSI)-4 and the Stuttering Severity Scale (SSS) (4). In PWS and controls, cognitive function and mood were assessed using the Mini-Mental State Evaluation (MMSE) (40), the Frontal Assessment Battery (FAB) scale (41) and the Hamilton depression scale (HAM-D) (42). Participant demographic and clinical features are reported in Table 1.

**Table 1:** Demographic and clinical characteristics of the participants.

	N (F, M)	Age (y)	Weight (kg)	Height (cm)	MMSE	FAB	HAM-D	SSS	SSI-4	VHI	LRs
PWS	53 (24, 29)	16.7 ± 7.6	51.6 ± 16.2	159.2 ± 17.6	29.5 ± 0.7	17.5 6 ± 0.8	0.6 ± 0.8	3.2 ± 1.7	20.5 ± 9.0	48.3 ± 22.32	0.84 ± 0.21
cPWS	20 (9, 11)	9.1 ± 1.6	34.5 ± 13.6	141.1 ± 15.1	29.7 ± 0.7	17.6 ± 0.7	1.1 ± 0.8	3.2 ± 1.6	20.0 ± 8.9	48.1 ± 21.7	0.62 ± 0.21
yPWS	33 (15, 18)	21.3 ± 5.8	61.9 ± 5.2	170.2 ± 6.1	29.4 ± 0.8	17.4 6 ± 0.9	0.4 ± 0.6	3.1 ± 1.8	20.8 ± 9.2	48.5 ± 22.8	0.6 ± 0.30
C	71 (29, 44)	16.2 ± 6.5	51.1 ± 16.2	157.5 ± 17.7	29.4 ± 0.8	17.4 ± 0.9	0.6 ± 0.8	–	–	–	–
cC	31 (12, 19)	9.6 ± 1.5	37.2 ± 1.4	140.1 ± 10.5	29.2 ± 0.9	17.3 ± 0.9	0.7 ± 0.9	–	–	–	–
yC	40 (17, 23)	21.1 ± 3.9	61.9 ± 10.7	170.6 ± 8.1	29.5 ± 0.7	17.5 ± 0.8	0.5 ± 0.7	–	–	–	–

N, number; C, the whole group of controls (children and younger adults); cC, controls in prepubertal age; yC, younger adult controls in postpubertal age; PWS, the whole group of people with stuttering; cPWS, children people with stuttering in the prepubertal age; yPWS, younger adult people with stuttering in the postpubertal age; FAB, frontal assessment battery; HAM-D, hamilton depression rating scale; MMSE, mini-mental state evaluation; SSI-4, stuttering severity instrument 4; SSS, stuttering severity scale; VHI, voice handicap index; LR, likelihood ratio. LR scores were calculated from the sustained emission of a vowel in people with stuttering. Results are expressed as average ± standard deviation.

#### 2.1.1. Audio recordings and machine-learning analysis

**Figure 1:**
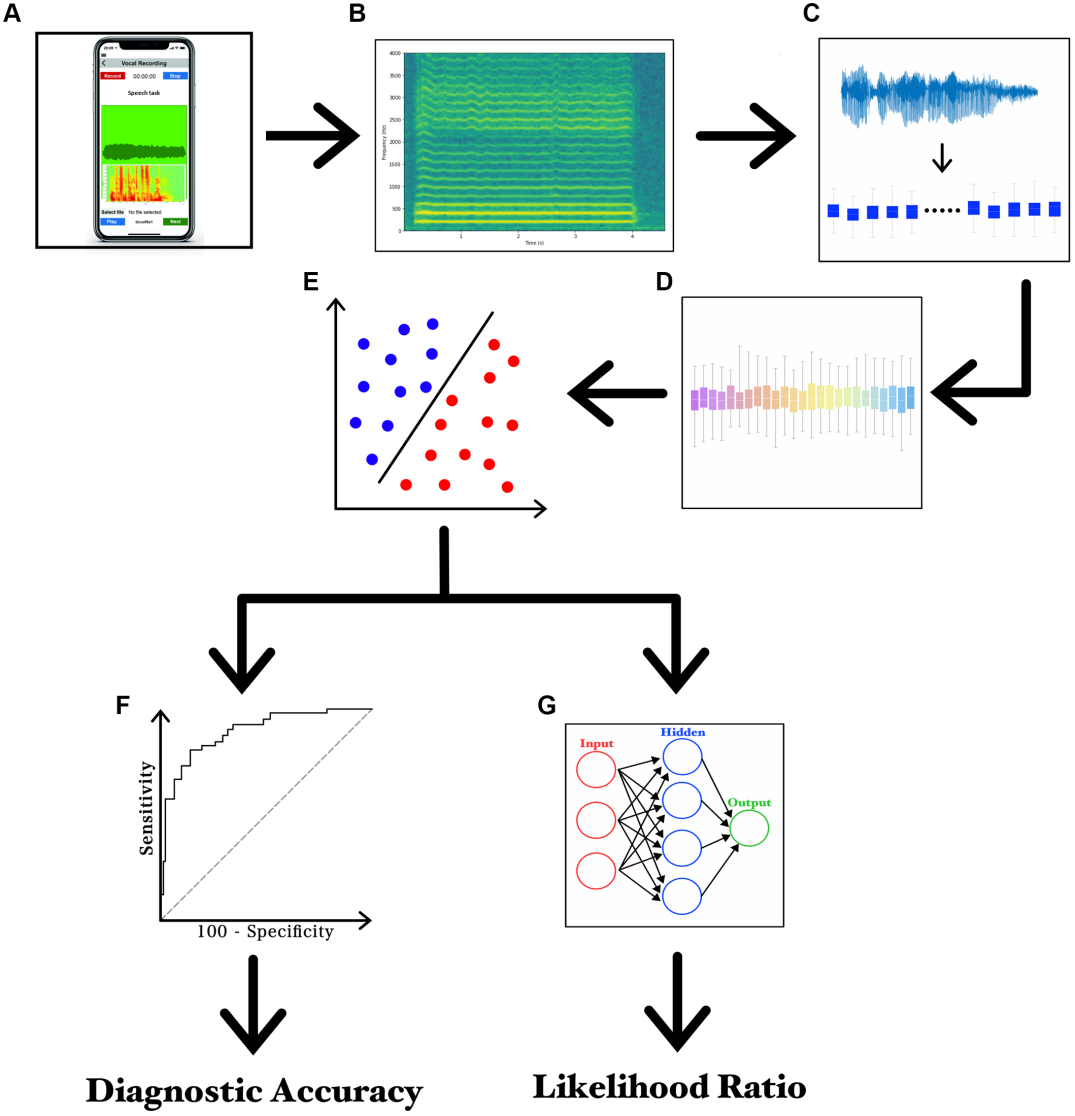
Experimental paradigm. **(A)** recording of voice samples using a smartphone; **(B)** narrow-band spectrogram of the acoustic voice signal; **(C)** feature extraction; **(D)** feature selection; **(E)** feature classification; **(F)** ROC curve analysis; and **(G)** LR values calculated through ANN.

The recording session started by asking participants to sit on a chair in the middle of a silent room, at home. By sending the written experimental paradigm *via* email, participants were instructed to handle and face a smartphone at about 30 cm from the mouth and then to speak with their usual voice intensity, pitch, and quality. For audio recordings, participants used smartphones currently available in the market (various brands including Apple^®^, Google^®^, Samsung^®^, Huawei^®^, Xiaomi^®^, and Asus^®^). For both patients and controls, the experimental design included a single recording session based on three separate speech tasks. The first speech task consisted of the sustained emission of the vowel/e/ for 5 s, whereas the second and third tasks consisted of the reading of samples of connected speech. More in detail, the second speech task was the following Italian phonetically balanced sentence: “*Nella casa in riva al mare maria vide tre cani bianchi e neri*” (s1) (29, 32), whereas the last speech task was a rich-in-occlusive-consonant Italian sentence: “*Poichè la principessa non tornava al castello, la regina ordinò alle guardie di cercarla nal bosco*” (s2), known to induce detrimental effects on stuttering. Audio recordings were collected according to a previously reported standardized procedure (29, 32, 43). To simplify the procedures of home-made audio recording, all participants were asked to save the audio tracks in mp4 format at the end of the recording session and then, to send audio tracks by encrypted e-mail to our institutional mail server, which was protected by password and accessible only by the authors. Lastly, a segmentation procedure was applied to separate each audio track into single recordings of speech samples (Audacity^®^) (29). The machine-learning analysis was based on specific and standardized algorithms, namely the SVM, in agreement with previous works by our group in the field (44–47). The classification analysis was based on SVM built with a linear kernel for binary classifications. Training of SVM classifier consisted of the first 30 most relevant features ranked by the CAE, in order to reduce the number of selected features needed to perform the classification and to reduce the probability of overfitting. A list of the first 30 features which represent functionals applied to audio LLDs – extracted from the vowel for the comparison between PWS and controls is reported in Table 2. A detailed discussion on technical issues concerning the analysis has been provided in Supplementary material 1. Also, the experimental paradigm is summarized in Figure 1 and Supplementary file 1.

**Table 2:** List of the first 30 selected features for the comparison between C and PWS during the sustained emission of a vowel.

No	Group	Family	Low level descriptor	Functional
1	Cepstral	Spectral LLD	MFCC 1–14	Standard deviation
2	Prosodic	Energy related LLD	Zero crossing rate	Relative peak range
3	Prosodic	Energy related LLD	Sum of auditory spectrum (loudness)	Relative minimum range
4	Prosodic	Energy related LLD	Zero crossing rate	Coefficient 0 of linear prediction
5	Cepstral	Spectral LLD	MFCC 1–14	Absolute peak range
6	Cepstral	Spectral LLD	MFCC 1–14	Relative peak mean
7	Spectral	Spectral LLD	Spectral Variance	Standard deviation of peak distances
8	Sound Quality	Voicing related LLD	Probability of voicing	Arithmetic mean
9	Prosodic	Energy related LLD	Zero crossing rate	Absolute peak range
10	Cepstral	Spectral LLD	MFCC 1–14	Absolute peak range
11	Cepstral	Spectral LLD	MFCC 1–14	99% percentile
12	Cepstral	Spectral LLD	MFCC 1–14	Range
13	Cepstral	Spectral LLD	MFCC 1–14	3^rd^ coefficient of linear prediction
14	Prosodic	Energy Related LLD	RMS Energy	Relative peak range
15	Cepstral	Spectral LLD	MFCC 1–14	Inter-quartile 1–2
16	Cepstral	Spectral LLD	MFCC 1–14	Standard deviation of rising slope
17	Spectral	Spectral LLD	RASTA-PLP style auditory spectrum, bands 1–26 (0–8 kHz)	Absolute peak range
18	Cepstral	Spectral LLD	MFCC 1–14	Inter-quartile 1–2
19	Cepstral	Spectral LLD	MFCC 1–14	99% percentile
20	Prosodic	Energy Related LLD	Sum of auditory spectrum (loudness)	Centroid
21	Spectral	Spectral LLD	Spectral Flux	3^rd^ coefficient of linear prediction
22	Prosodic	Voicing Related LLD	Fundamental frequency	Relative duration of the LLD is above 25%
23	Spectral	Spectral LLD	RASTA-PLP style auditory spectrum, bands 1–26 (0–8 kHz)	Relative peak mean
24	Cepstral	Spectral LLD	MFCC 1–14	Absolute peak range
25	Spectral	Spectral LLD	RASTA-PLP style auditory spectrum, bands 1–26 (0–8 kHz)	Mean of peak distances
26	Spectral	Spectral LLD	Spectral Skewness	Absolute peak range
27	Cepstral	Spectral LLD	MFCC 1–14	Absolute peak mean
28	Sound Quality	Voicing related LLD	Jitter (Delta/Derivative)	2^nd^ coefficient of linear prediction
29	Spectral	Spectral LLD	Spectral variance	Relative peak range
30	Cepstral	Spectral LLD	MFCC 1–14	2^nd^ coefficient of linear prediction

C, the whole group of controls; PWS, the whole group of people with stuttering; LLD, low level descriptor, MFCC, mel-frequency spectral coefficients; RASTA-PLP, relative spectral transform-perceptual linear prediction.

### 2.2. Statistical analysis

The normality of the demographic and anthropometric variables in patients and controls was assessed using the Kolmogorov-Smirnov test. Mann–Whitney U test was used to compare demographic scores in patients and controls. ROC analyses were performed to identify the optimal diagnostic cut-off values of the support vector machine (SVM) (selected features), for discriminating between:

1. controls vs. PWS during the sustained emission of the vowel and sentence s1;
2. controls vs. PWS during the emission of sentences s1 and s2;
3. yC vs. yPWS during the sustained emission of the vowel and sentence s1;
4. cC vs. cPWS during the sustained emission of the vowel and sentence s1;
5. cC vs. yC during the sustained emission of the vowel and sentence s1;
6. cPWS vs. yPWS during the sustained emission of the vowel and sentence s1.

Cut-off values were calculated as the point of the curves with the highest Y.I. (sensitivity + specificity −1) to maximize the sensitivity and specificity of the diagnostic tests. The positive and negative predictive values were also calculated. According to standardized procedures (48), we compared the area under the curves (AUCs) in the ROC curves calculated from SMO (selected features) to verify the optimal test for discriminating within the subgroups. All ROC analyses were performed using MATLAB. Spearman’s rank correlation coefficient was used to assess correlations between clinical scores and LR values. *p* < 0.05 was considered statistically significant. Unless otherwise stated, all values are presented as mean ± standard deviation (SD). Statistical analyses were performed using Statistica version 10 (StatSoft, Inc) and MATLAB (Mathworks, Inc.).

## 3. Results

The Kolmogorov–Smirnov test showed that demographic (age) and anthropometric (weight and height) parameters were normally distributed in controls and people with stuttering (*p* < 0.05). Mann-Whitney U test showed comparable demographic, MMSE, FAB, and HAM-D scores in patients and controls (*p* > 0.05) (Table 1). The clinical assessment of stuttering was based on VHI, SSI-4, and SSS scales. AVHI mean ± SD score of 48.3 ± 22.32 is suggestive of moderate disease severity; an SSI-4 mean ± SD score of 20.5 ± 9.0 is indicative of a mild-to-moderate stuttering severity both in children and adults; An SSS mean ± SD score of 3.2 ± 1.7 is suggestive of mild disease severity (49). The recorded MMSE, FAB, and HAM-D scores in both PWS and controls suggest normal cognition and the absence of mood depression (All *p* values >0.05; Table 1).

### 3.1. Machine-learning analysis

**Figure 2:**
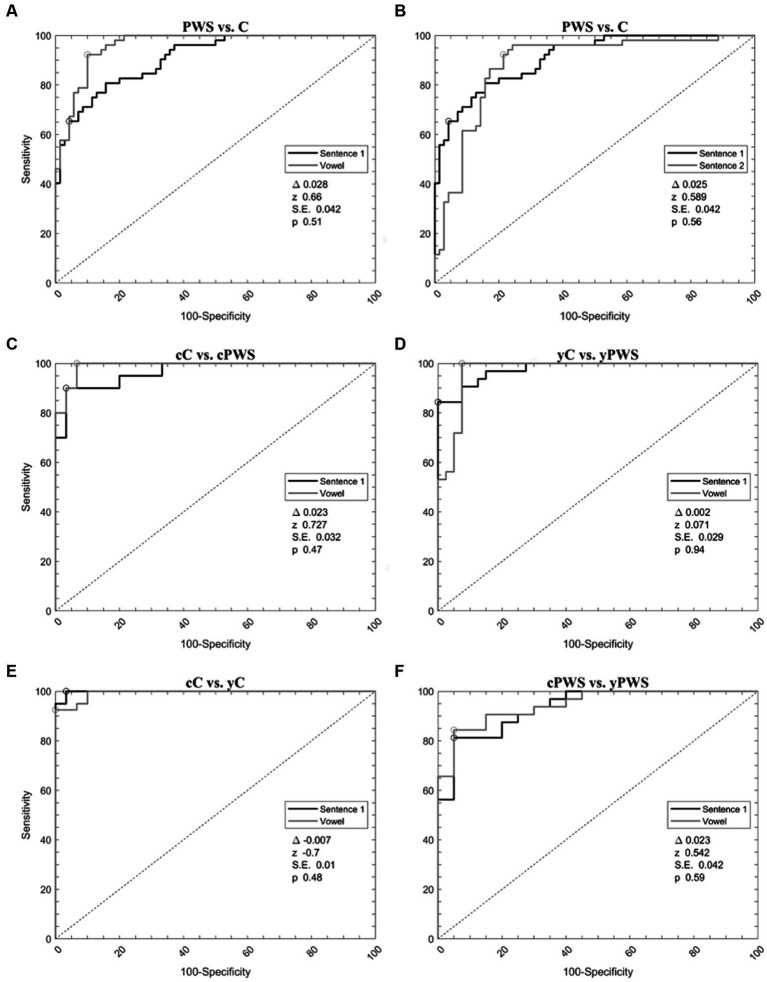
Support vector machine analysis of voice samples. Receiver operating characteristic curves calculated with a support vector machine to differentiate: (1) Controls and people with stuttering during the emission of vowel and sentence 1 **(A)**; (2) Controls and people with stuttering during the emission of sentence s1 and s2 **(B)**; (3) Younger adult controls and people with stuttering during the emission of vowel and sentence 1 **(C)**; (4) Children controls and people with stuttering during the emission of vowel and sentence 1 **(D)**; (5) Children and younger adult controls during the emission of vowel and sentence s1 **(E)**; (6) Children and younger adult people with stuttering during the emission of vowel and sentence s1 **(F)**. C, the whole group of controls; cC, controls in prepubertal age; yC, younger adult controls in postpubertal age; PWS, the whole group of people with stuttering; cPWS, children with stuttering in the prepubertal age; yPWS, younger adults with stuttering in the postpubertal age.

When analyzing PWS versus controls during the sustained emission of the vowel, ROC curve analyses identified an optimal diagnostic threshold value of −0.24 (associated criterion) with Y.I. of 0.75. Furthermore, during the sustained emission of the sentence s1, ROC curve analyses identified an optimal diagnostic threshold value of 0.12 with a Y.I. of 0.66. Then, we compared the ROC curves obtained during the emission of the vowel and the sentence s1 showing comparable results (the difference between AUCs = 0.028, *z* = 0.660, SE = 0.042, and *p* = 0.51) (Figure 2A, Table 3).

**Table 3:** Performance of the machine-learning algorithm.

Comparisons	Speech-task	Assoc. criterion	Youden index	Se (%)	Sp (%)	PPV (%)	NPV (%)	Acc (%)	AUC
PWS vs. C	Vowel	−0.24	0.75	88.7	86.3	90.0	84.6	87.7	0.934
	S1	0.12	0.66	85.7	80.8	85.7	80.8	83.6	0.906
	S2	−0.09	0.62	81.3	80.9	87.1	73.1	81.1	0.881
cC vs. cPWS	Vowel	0.23	0.94	93.8	100	100	90.0	96.0	0.993
	S1	0.06	0.85	90.6	94.4	96.7	85.0	92.0	0.970
yC vs. yPWS	Vowel	0.66	0.80	92.3	87.9	90.0	90.6	90.3	0.975
	S1	0.07	0.81	88.4	93.1	95.0	84.4	90.3	0.973
cC vs. yC	Vowel	0.39	0.88	93.3	95.5	93.3	95.5	94.3	0.991
	S1	0.43	0.94	96.7	97.5	96.7	97.5	97.1	0.998
cPWS vs. yPWS	Vowel	0.20	0.86	94.4	91.2	85.0	96.9	92.3	0.956
	S1	0.24	0.89	100	88.9	80.0	100	92.3	0.933

C, the whole group of controls; cC, controls in prepubertal age; yC, younger adult controls in postpubertal age; PWS, the whole group of people with stuttering; cPWS, children people with stuttering in the prepubertal age; yPW, younger adult people with stuttering in the postpubertal age; Acc, accuracy; AUC, area under the curve; NPV, negative predictive value; PPV, positive predictive value; Se, sensitivity; Select Sent., selective sentence which consists of the emission of the sentence of the connected speech supposed to induce stuttering in patients; Sp, specificity; Stand. Sent., standard sentence which is the sentence of the connected speech used in our previous papers in the field of voice analysis. Instances refer to the number of subjects considered in each comparison. Cross-validation refers to standardized procedures of a machine-learning algorithm (see the text for details).

Machine-learning discriminated between PWS and controls during the emission of the sentence s1 and s2. When comparing the 30 most relevant selected features extracted from the emission of s2, ROC curve analyses identified an optimal diagnostic threshold value of −0.09 with Y.I. of 0.62. Then, we compared the ROC curves obtained during the emission of s1 and s2 showing comparable results (the difference between AUCs = 0.025, *z* = 0.589, SE = 0.042, and *p* = 0.56) (Figure 2B, Table 3).

Concerning the classification of cC vs. cPWS during the sustained emission of the vowel and sentence s1, for the emission of the vowel, ROC curve analyses identified an optimal diagnostic threshold value of 0.23 with YI of 0.94. Furthermore, during the sustained emission of the sentence s1, ROC curve analyses identified an optimal diagnostic threshold value of 0.06, with YI of 0.85. Then, we compared the ROC curves obtained during the emission of the vowel and sentence s1 showing comparable results (the difference between AUCs = 0.023, *z* = 0.727, SE = 0.032, and *p* = 0.47) (Figure 2C, Table 3).

The discrimination between yC vs. yPWS, during the sustained emission of the vowel and sentence s1 also disclosed high accuracy. When comparing the 30 related most relevant selected features extracted from the emission of the vowel, ROC curve analyses identified an optimal diagnostic threshold value of 0.66 (associated criterion), when applying discretization and 10-folds cross-validation (Y.I. = 0.80). Furthermore, when comparing 30 selected features extracted from the sustained emission of the sentence s1, ROC curve analyses identified an optimal diagnostic threshold value of 0.07, when applying discretization and 10-folds cross-validation (Y.I. = 0.81). Afterwards, we compared the ROC curves obtained during the emission of the vowel and sentence s1 showing comparable results (the difference between AUCs = 0.002, *z* = 0.071, SE = 0.029, and *p* = 0.94) (Figure 2D, Table 3).

When comparing cC and yC with sequential minimal optimization (SMO), we were able to obtain high results for both the emission of the vowel and sentence s1. For the emission of the vowel, ROC curve analyses identified an optimal diagnostic threshold value of 0.39 with Y.I. of 0.88. Furthermore, when comparing 30 selected features extracted from the sustained emission of the sentence s1, ROC curve analyses identified an optimal diagnostic threshold value of 0.43, when applying discretization and 10-folds cross-validation (Y.I. = 0.94). Then, we compared the ROC curves obtained during the emission of the vowel and sentence s1 showing comparable results (the difference between AUCs = −0.007, *z* = −0.700, SE = 0.010, *p* = 0.48) (Figure 2E, Table 3).

Finally, we discriminated between cPWS and yPWS with SMO. In this case, we were also able to obtain high results for both the emission of the vowel and sentence s1. For the emission of the vowel, ROC curve analyses identified an optimal diagnostic threshold value of 0.20 (associated criterion) with Y.I. of 0.86. Furthermore, during the sustained emission of the sentence s1, ROC curve analyses identified an optimal diagnostic threshold value of 0.24, with a Y.I. of 0.89. Then, we compared the ROC curves obtained during the emission of the vowel and sentence s1 showing comparable results (the difference between AUCs = 0.023, *z* = 0.542, SE = 0.042, and *p* = 0.59) (Figure 2F, Table 3).

### 3.2. Correlation analysis

In the group of PWS, the Spearman test disclosed a negative correlation between age and HAM-D (*r* = −0.35, *p* = 0.012), i.e., young PWS manifest a higher psychological burden of disease. Also, we found highly relevant positive correlations among scores at clinical scales for the multidimensional assessment of stuttering. More in detail, VHI significantly correlated with SSS (*r* = 0.95, *p* < 0.01) and SSI-4 (*r* = 0.97, *p* < 0.01), i.e., the higher impairment of stuttering, the greater the stuttering-related complaint. Lastly, SSS positively correlated with SSI-4 (*r* = 0.94, *p* < 0.01).

Concerning the clinical-instrumental correlations, we found a positive correlation between LRs collected in the overall group of PWS during the sustained emission of vowel/e/ as well as during the reading of sentences S1 and S2 and SSS (*r* = 0.31, *p* = 0.02, for the vowel; *r* = 0.30, *p* = 0.03, for S1; *r* = 0.44, *p* < 0.01, for S2), SSI-4 (*r* = 0.32, *p* = 0.02, for the vowel; *r* = 0.36, *p* < 0.01, for S1; *r* = 0.46, *p* < 0.01, for S2) and VHI (*r* = 0.32, *p* = 0.02, for the vowel; *r* = 0.35, *p* = 0.01, for S1; *r* = 0.43, *p* < 0.01, for S2). The higher the LR values attributed by machine-learning, the higher disability and severity of overall stuttering and voice symptoms. When considering subgroups of cPWS as well as yPWS, we reported a higher positive correlation between LRs calculated during the emission of speech tasks and scores at clinical scales (i.e., SSS, SSI-4, and VHI). More in detail, in cPWS and yPWS, LRs positively correlated with SSS during the emission of the vowel (*r* = 0.67, *p* < 0.01, and *r* = 0.43, *p* = 0.02, respectively), S1 (*r* = 0.53, *p* = 0.02, and *r* = 0.54, *p* < 0.01, respectively) and S2 (*r* = 0.67, *p* < 0.01, and *r* = 0.42, *p* = 0.02, respectively). Still, in cPWS and yPWS, LRs positively correlated with SSI-4 during the emission of the vowel (*r* = 0.63, *p* < 0.01, and *r* = 0.54, *p* < 0.01, respectively), S1 (*r* = 0.56, *p* = 0.02, and *r* = 0.51, *p* < 0.01, respectively) and S2 (*r* = 0.62, *p* < 0.01, and *r* = 0.42, *p* = 0.02, respectively). Lastly, in cPWS, LRs also positively correlated with VHI during the emission of the vowel (*r* = 0.64, *p* < 0.01, and *r* = 0.51, *p* < 0.01, respectively), S1 (*r* = 0.57, *p* = 0.02, and *r* = 0.50, *p* < 0.01, respectively) and S2 (*r* = 0.61, *p* < 0.01, and *r* = 0.41, *p* = 0.02, respectively).

## 4. Discussion

In this study, we objectively recognized people with stuttering by using artificial intelligence (i.e., the SVM algorithm). We identified relevant acoustic features altered in stuttering, achieving high classification accuracy in discriminating between PWS and controls. We also found a significant effect of ageing in modifying abnormal acoustic features reported in stuttering, as shown by high classification accuracy when discriminating between independent ageing groups, among PWS and controls. Our analysis was highly consistent and reliable as suggested by the lack of linguistic-related detrimental effects exerted by speech tasks on stuttering. Lastly, we found significant clinical-instrumental correlations pointing to the great medical relevance of our analysis. Overall, our findings support the role of machine learning in the objective recognition of specific voice disorders (50–52).

To collect homogeneous audio recordings in PWS and controls, we carefully controlled for several methodological factors. Participants were all native Italian speakers, non-smokers, and did not report pathologic conditions affecting voice emission, thus allowing for the exclusion of possible confounding factors. All young adults (e.g., both people with stuttering and controls) completed the pubertal development, thus excluding incomplete development of systems involved in voice emission. Patients with stuttering had similar demographic and anthropometric characteristics (e.g., height, weight, and BMI) as compared with controls, thus excluding confounding related to these physiologic factors. Subjects with cognitive impairment and mood disorders were excluded from the study cohort. Furthermore, since the worsening of dysfluencies during the day, we asked participants to record audio samples in the morning, when possible. The stuttering diagnosis was based on current international guidelines. As speech tasks, we selected the sustained emission of a vowel and sentences according to standardized procedures, respectively. All samples were recorded through smartphones currently available on the market and able to save audio tracks in the required file format. Corrupted recordings were excluded from the analysis.

### 4.1. Clinical evaluation In people with stuttering

Clinically, both cPWS and yPWS showed mild-to-moderate disease severity in terms of stuttering, and absent depression, thus confirming developmental stuttering. The mild-to-moderate stuttering severity measured in our sample of PWS is in line with the literature findings of a full recovery during the first four years of age reported in up to 75% of pre-schoolers with developmental stuttering (2, 53), thus showing a milder form of the disease once grown-up. We found that young PWS manifest a trend towards a higher psychological burden of disease, though without reaching statistical significance. Also, we found highly relevant positive correlations among scores at clinical scales for the multidimensional assessment of stuttering (VHI, SSS, and SSI-4), independently from the specific age groups.

### 4.2. Machine-learning analysis in people with stuttering

In this study, we objectively recognized PWS by acoustic analysis based on SVM. This finding is highlighted by the high reliability and accuracy of results achieved during the emission of speech tasks when classifying controls and PWS. As shown by the most relevant acoustic features selected by machine-learning, our methodology fits with previous studies based on spectral analysis, thus confirming the biological plausibility of our observations. It should be mentioned that all previous studies aiming at the objective analysis of stuttering were based on standardized spectral analysis and were able to find multiple abnormal acoustic features in stuttering (13–26). These investigations have certainly contributed to improve current knowledge of stuttering by reporting specific changes in acoustic features (13–26). However, standard acoustic analysis does not allow for dynamically combining selected features extracted from a large dataset, and it does not offer the opportunity to automatically learn and improve from experience (29, 32, 33). Therefore, other authors have applied automatic tools to detect speech impairment in the field of stuttering, for classification purposes. Historically, the first attempt to automatically and objectively detect stuttering through machine-learning comes from the automatic speech recognition (ASR) analysis, consisting of a preliminary process that converts audio signals to text, and then a second phase used to detect and identify linguistic abnormalities related to stuttering (54–56). The main limitation of this approach was the great number of errors. Therefore, authors have begun to apply machine-learning to acoustic features extracted from audio recordings in patients with stuttering, using several approaches including but not limited to the Mel frequency cepstral coefficients (MFCC), the ANN, the hidden Markov model (HMM), and finally the SVM (57–62). Despite the achievements in the objective analysis of stuttering, previous research in the field was characterized by several limitations. Machine-learning studies in stuttering have included small and heterogeneous cohorts of audio samples collected in a dataset of patients with stuttering. Also, the large part of datasets of audio recordings from stutterers lacks relevant clinical as well as anthropometric parameters which are known to be involved in voice emission. Also, it has been documented that PWS show difficulties in coordinating airflow, articulation, and resonance, and minor asynchronies have been found even during fluent speech (63). Hence, we believe that our study demonstrates the ability of acoustic analysis to objectively recognize PWS. The acoustic features selected by our classifier and used for further classification purposes correspond to those considered in previous seminal works in the field, using spectral and cepstral analysis (see also Table 2) (57–62). Moreover, our study showed for the first-time significant clinical-instrumental correlations: the higher the LR values attributed by machine-learning, the higher severity of symptoms in PWS. Hence, we demonstrated that the degree of voice changes in PWS correlates with disease severity, and finally, LR values can be considered reliable scores to express the severity of PWS.

Our machine-learning approach would further help in clarifying the pathophysiological underpinning of stuttering. Recent clinical and experimental observations have raised the hypothesis that stuttering reflects impaired sensorimotor integration in specific brain networks (64, 65), to the point that it may be even considered as a form of focal/segmental action dystonia (66, 67). Hence, we conjecture that future experimental investigations combining machine-learning analysis of voice with neurophysiological and neuroimaging techniques would help in better assessing voice-related changes in sensorimotor integration in PWS (34).

### 4.3. Effect of ageing in people with stuttering

Concerning the effect of ageing on acoustic features in stuttering, this is the first study to demonstrate a significant role of ageing in changing acoustic features known to be altered in stuttering as shown by previously unreported high accuracy in classifying children (i.e., 7–12 years) and younger adults (i.e., 15–30 years) among PWS and controls. The observation that machine-learning can achieve high classification accuracy when discriminating between cC and yC allows us to confirm and expand data reported in a previous publication by our groups on healthy controls in which we showed that objective acoustic analysis distinguished between younger and older adults, with a high level of accuracy (29). In this study, we demonstrated a similar effect of human ageing on acoustic features in PWS, as suggested by the high accuracy when classifying cPWS and yPWS. As previously demonstrated for healthy controls, similar age-related changes in physiological functions may explain our findings in PWS. More in detail, the physiological basis underlying our results is prominently linked to age-related changes in the phonatory apparatus, including the effects of hormone molecules during the transition from pre-pubertal to post-pubertal age. Still, we found significant clinical-instrumental correlations also in cPWS and yPWS: the greater the LR values, the higher severity of PWS. Interestingly, when classifying cC and cPWS as well as yC and yPWS we obtained higher results than those achieved during the comparison between PWS and the overall group of controls. These results confirm previous data reported by our group showing that the accuracy of the machine-learning algorithm tended to further improve when comparing the groups of subjects with a narrower age band (29).

### 4.4. Effect of speech task in people with stuttering

We observed similar classification accuracy for each speech task considered in the analysis, namely the sustained emission of the vowel and sentences. This issue could be possibly judged controversial since it is known that several types of dysfluencies in PWS, including involuntary, audible, or silent, repetitions or prolongations of sounds, syllables, words, sentences, dysrhythmic phonation, such as blocks, and prolongations tend to worsen in response to specific speech task (i.e., the detrimental effect of linguistic issues). However, as already reported for other disorders affecting voice, including laryngeal dystonia and Parkinson’s disease, we speculate that machine learning is able to detect subtle changes in acoustic features occurring even during a simple voice task (i.e., the sustained emission of a vowel). This finding is of great relevance since it testifies the transveral applicability of machine learning analysis among different languages. With the present study we showed the wide applicability of our analysis for telemedicine purposes, as shown by the results achieved using smartphone-recorded audio samples, in an ecologic scenario. The importance of the application of telemedicine to research on stuttering has been previously suggested, although with some technical limitations (68), which have been resolved in the present study. It is well-known that telemedicine and telepractice can be used as complementary therapeutic methods for neurologic diseases in general (69), and for stuttering in particular (68), hence we are confident that our study will lay the foundation for future therapeutic efforts in this field.

## 5. Conclusion

We achieved high classification accuracy when discriminating between PWS and controls. We also demonstrated a significant effect of ageing, as shown by high accuracy when discriminating between children and younger controls as well as PWS. Furthermore, we showed that our analysis was highly consistent and reliable as suggested by the lack of linguistic-related detrimental effects on stuttering. In addition, we demonstrated the applicability of our analysis for telemedicine purposes. Lastly, we found significant clinical-instrumental correlations pointing to machine-learning analysis as a consistent and reliable tool to objectively diagnose stuttering. To date, therapeutic strategies for stuttering mostly rely on non-pharmacological approaches based on speech therapy or on stuttering devices, which alter the voice frequency or slow the rate of speech using auditory feedback (53). Within this clinical and therapeutic context, we believe that acoustic analysis could be highly useful for checking and monitoring PWS over time (e.g., before and after a given therapeutic approach). This aspect is valuable and innovative, due to the significant lack of studies on the objective assessment of treatment outcomes in PWS. Ultimately, we are confident that future studies using machine-learning techniques and automated acoustic analysis, may further help clinicians in the classification and management of stuttering as well as other neurodevelopmental disorders of speech production (53).

## Data availability statement

The original contributions presented in the study are included in the article/Supplementary material, further inquiries can be directed to the corresponding author.

## Ethics statement

The studies involving human participants were reviewed and approved by Institutional Review Board (0026508/2019). Written informed consent to participate in this study was provided by the participants’ legal guardian/next of kin.

## Author contributions

FA and LM: organization, execution of the study design, writing of the first draft, review, and critique. AS and GS: conception, organization of the study design, review, and critique. EM, PDL, and MP, execution of the study design, writing of the first draft, review, and critique. LL, GR, FDG, and DT: review and critique. GC: conception, organization of the study design, review, and critique. All authors contributed to the article and approved the submitted version.

## Conflict of interest

LM has received honoraria from the International Association of Parkinsonism and Related Disorders (IAPRD) Society for social media and web support and a grant contribution from the International Parkinson Disease Movement Disorders Society for the Unified Tremor Rating Scale Validation Program.

The remaining authors declare that the research was conducted in the absence of any commercial or financial relationships that could be construed as a potential conflict of interest.

The reviewer FM declared a past collaboration with the authors LM at the time of the review.

## Publisher’s note

All claims expressed in this article are solely those of the authors and do not necessarily represent those of their affiliated organizations, or those of the publisher, the editors and the reviewers. Any product that may be evaluated in this article, or claim that may be made by its manufacturer, is not guaranteed or endorsed by the publisher.
