# Supplementary material for: Acoustic analysis in stuttering: a machine-learning study
Source: Front Neurol. 2023 Jun 30;14:1169707. doi: 10.3389/fneur.2023.1169707 (PMC10347393; doi:10.3389/fneur.2023.1169707)
Supplement: Supplementary file 1 [file Data_Sheet_1.docx]

**Supplementary file 1: Machine-learning analysis**

The machine-learning analysis was implemented by applying specific and standardized algorithms of artificial intelligence (1-4). We converted all the audio tracks from mp4 into Wav format (sampling frequency: 44.1 kHz; bit depth: 16 bit), before submitting data to OpenSMILE, a dedicated software for the pre-process of feature extraction (OpenSMILE; audEERING GmbH, Germany) (2, 5). For each voice sample, 6139 voice features were extracted by using a modified INTERSPEECH2016 Computational Paralinguistics Challenge (IS ComParE 2016) feature dataset (5). IS ComParE 2016 contains voice features calculated using computational functionals (e.g., mean, quartiles, percentiles, position of max/min, linear regression) over acoustic low-level descriptors (LLDs), including those related to the energy, spectrum, cepstrum of the signal (5), and also including the Mel-Frequency Cepstral Coefficients (6), RASTA-PLP Coefficients (7), jitter, shimmer, sound quality descriptors, and prosodic features.

To identify a small subset of relevant features for the objective analysis of stuttering, the extracted voice features underwent feature selection using the correlation features selection (CFS) algorithm (8). Through CFS, we selected voice features highly correlated with the class, thus removing the irrelevant and redundant features from the original dataset. Selected features were ranked by using the correlation attribute evaluation (CAE) algorithm, which evaluates and ranks all the attributes in order of relevance, according to Pearson’s correlation method.

After pre-processing procedures, we prepared the classification analysis, by training a support vector machine (SVM) classifier based on linear kernel, in order to achieve binary classifications. To train the SVM classifier, we considered only the first 30 most relevant features ranked by the CAE, in order to reduce the number of selected features needed to perform the classification and to reduce the probability of overfitting. A list of the first 30 features which represent functionals applied to audio LLDs - extracted from the vowel for the comparison between PWS and C is reported in Table 2.

Specifically, the SVM was trained using the sequential minimal optimization (SMO) method, which is considered a fast and efficient machine learning algorithm for implementing an SVM classifier (9). Given the limited number of instances (voice samples) contained in the dataset, to obtain more reliable results, all the classifications were made using a 10-folds cross-validation procedure. The outcome of classification analysis consisted of ROC curves and statistics (such as Accuracy, Specificity, Sensitivity, etc…), as clarified in the “Statistical analysis” section. Both procedures of selection and classification were performed through Weka, a dedicated software containing a collection of algorithms for data analysis and predictive modelling (Weka, Waikato Environment for Knowledge Analysis, University of Waikato, New Zealand) (8, 10).

Moreover, we used a feed-forward artificial neural network (ANN), consisting of a 30-neurons input layer, a 10-neurons hidden layer and a one-neuron output layer, for clinical-instrumental correlation purposes. Input for ANN consisted of the first 30 most relevant selected features, which thus matched the 30-neurons input layer. Then, the ANN was trained to calculate a continuous numerical value (the likelihood ratio - LR), ranging from 0 to 1 and reflecting the degree of voice impairment in each patient with PD (i.e. the closer the LRs to 1, the higher the degree of voice impairment). ANN was trained by using the same selected features used to train the SVM.

**References**

1. E. A. Introduction to machine learning. In: Press M, editor. Cambridge, Mass.2010.

2. Eyben F WM, Schuller B. . Opensmile: the munich versatile and fast open-source audio feature extractor. In: Press A, editor. Proceedings of the international conference on Multimedia. Firenze, Italy2010. p. 1459.

3. Russell SJ NP, Davis E. . Artificial intelligence: a modern approach. In: Hall USRP, editor. 3rd Edition ed2010.

4. Specht DF. A general regression neural network. IEEE Trans Neural Netw. 1991;2(6):568-76.

5. Schuller B SS, Batliner A, Hirschberg J, Burgoon JK, Baird A, Elkins A, Zhang Y, Coutinho E, Evanini K. The INTERSPEECH 2016 Computational Paralinguistics Challenge: Deception, Sincerity and Native Language. 2016. p. 2001–5.

6. Davis S MP. Comparison of parametric representations for monosyllabic word recognition in continuously spoken sentences. IEEE Transactions on Acoustics, Speech, and Signal Processing. 281980. p. 357–66.

7. Ganapathy S, Thomas S, Hermansky H. Modulation frequency features for phoneme recognition in noisy speech. J Acoust Soc Am. 2009;125(1):El8-12.

8. Hall M FE, Holmes G, Pfahringer B, Reutemann P, Witten IH. . The WEKA data mining software: an update. ACM SIGKDD Explorations Newsletter. 2009;11:10.

9. J. P. Probabilistic outputs for support vector machines and comparisons to regularized likelihood methods. Advances in Large Margin Classifiers. 1999;10:61–74.

10. Frank E HM, Holmes G, Kirkby R, Pfahringer B, Witten IH, Ian H. Weka-a machine learning workbench for data mining. In: Springer, editor. Data mining and knowledge discovery handbook2009. p. 1269–77.
